# Supplementary material for: Patterns of Intron Gain and Loss in Fungi
Source: PLoS Biol. 2004 Nov 30;2(12):e422. doi: 10.1371/journal.pbio.0020422 (PMC532390; doi:10.1371/journal.pbio.0020422)
Supplement: Table S1 — Also available at http://genes.mit.edu/NielsenEtAl/. (4.3 MB ZIP). [file pbio.0020422.st001.zip › NielsenEtAl/html/1435.html]

AN0225.1.NCU03591.1.MG04855.1.FG09017.1


```
 CLUSTAL W (1.82) Multiple Sequence Alignments - Introns Inserted


Sequence 1: NCU03591.1	1704 aa
Sequence 2: MG04855.1	1668 aa
Sequence 3: FG09017.1	1659 aa
Sequence 4: AN0225.1	1667 aa
Alignment Length: 1739 aa
Number Identitical Residues: 675 aa
Alignment Score (without introns) 35927


MG04855.1 	MGGVGQCQWPVWQVDDLTPCFQHD2YLRILLPAVVIGLSVLNLGFRSARHAAS--RSKS2
NCU03591.1	MG-FAQCGWPIWRTDDFTPCFQED2YLKILLPLIVVGLSLLRLLTSNLLRAAK--LKPN~
FG09017.1 	MG-------------------FAR2YLQILFPLIALFLSVALIAWTTITRAFSSWSKSK~
AN0225.1  	--MIPQCAGPIWAVDDLSRCFQRR2YLQEILPLAACALSLILITTRVAYRYIFS--RKS~
          	     ..  .    .. : .     **: ::*  .  **:  :      :   :    . 

MG04855.1 	RPGAHRTDISPDDDAIAQDDE-----DDDEGLAIGGGRLALVKTATKGSIVQADTPPAQT
NCU03591.1	HGYDRVADNEQDRTYLPRDEEPVDESDEDEALTMNGGRLALAKVTTKGSIVQADTPAGQT
FG09017.1 	ARYQQLSTEDRRHSHTDLPPEQVDDSEDDDALEINGGRLALVKTTTRGSIVQSDTPPGQL
AN0225.1  	FAYKLLPSDDVG-SVQDIRGAEEDLTEESDPMLSKAARIELP-------HFELDRPRGEI
          	      .  .             . :::.: :   ..*: *         .: * * .: 

MG04855.1 	LSVVVEELAIAGLVAVYVIALLSPKAHGSYTLTGTIIGLTTWVYVLVLATLRLFLGNTQW
NCU03591.1	ALTVIEILAIAGLVGVNVVALVLEAYSGHHGKIAAIAGLSAWTYVLILAALRLILRNTQW
FG09017.1 	ISQIIEELAIAGLVAVNVIQLSS-GSKGRHSRAAAITGLVIWVYVFVLSTLRLILGALKW
AN0225.1  	AFVVLEAAALVGQVVVFALVLKS-DAWGRDGRLPAAARLVSWSYLLFLVLVRLLLSVRQL
          	   ::*  *:.* * * .: *      *      :   *  * *::.*  :**:*   : 

MG04855.1 	RVPH-LWNHTAAIYSCQWLFLIGIFRSAMVHP-SSKLAQILVIVEFALTSLLFFMAITTR
NCU03591.1	RASH-LWNHTAAIYSAQWLFSLIGFRSVIVHP-ATQLRQTLTIVDFALTSLLFFMSISTR
FG09017.1 	RGPH-LWNHTAFLYSFNWVISVFLFRSAFLQDGISQVDQVTTFVEFGLITLLFGMAMTTR
AN0225.1  	HSPARLWNHTATLYGLQWLLTVLVFRSAVIYP-ISKRAMTFSSIEFSLSSFLVLLSVTTR
          	: .  ****** :*. :*:: :  ***..:    ::       ::*.* ::*. ::::**

MG04855.1 	KGNKTVLLEWEDGIPPARENLASLFSSFTFSWVDQIVWQGYKEPFEMGKVWNLLPKDKAA
NCU03591.1	KGNKTVILEWEDGIQPSRENLASLFSHFTFGWVDSIMWEGWKEPLTADRVWNLLPTDKAA
FG09017.1 	KGNKTVLLEWEDGIEPSREPLASLFSLAVFGWVDAIVYKGWKAPMEMEHVWNLLPKDKAA
AN0225.1  	RGNKPVLVPHEEGLEPARHPVASLLSLATFSWLDPLIFKGYRQSLELEDVWNLTASQKAS
          	:***.*::  *:*: *:*. :***:*  .*.*:* ::::*:: .:    **** ..:**:

MG04855.1 	TVLSHYRRVKKTTKLYWHLLRYFKGDLLSQAGWAVMSGMFTFAPTMLLKAILEYVE----
NCU03591.1	TVLANYRQVQKTTSLAYHLLSYFKGMVAAQAAWAIMSGAFTFAPTLLLKAILEYVE----
FG09017.1 	AVIADYRQMKRTGSLAMHLLKYFKRDLLIQCALAVLAGLFTFAPTLLLKVILEFVENYGT
AN0225.1  	TVLEDFRCRQYKGPLVVKLLRYFAGTILLQGAWTIFSNLFTYLPTLLLKAILEYVE----
          	:*: .:*  : .  *  :** **   :  * . ::::. **: **:***.***:**    

MG04855.1 	-EPESAPRSVVWLYVILLPVTDIIRSLGDNRALWIGRKICINVRAILVGEIYAKALRRKA
NCU03591.1	-DPTQAPRNVLWLYVILLPITDIVRSVGDAQALWIGRKICINIRAILVGEIYAKALRRKA
FG09017.1 	VSENDRPINVLWLYVIGLPVVDLIRSYADNQALWIGRKICIRVRALIIGDIYAKALRRKA
AN0225.1  	-DPRSTTPNAAWLYAVLLFVSAATQGVADGQALWIGRRMGVKLRAIIIGEIYAKALRRKA
          	 .  . . .. ***.: * :    :. .* :******:: :.:**:::*:**********

MG04855.1 	ATGKDTVLGSEKKEDKP-----KGGFISKIKKMLCLGDNDE-------SEDGKDGDKDKE
NCU03591.1	AAGNDKVLGEDKKDEAPA----KKGFCAKLKRALGLAKDDKKANDQSAAETAVTKAEDKA
FG09017.1 	AAGKDKVLLDEDKPKSTKGDEAPDGFMGKVKRALGMKDDKKATPNDAESANDSIKDDKKD
AN0225.1  	GPSVEAKKDEEKVPDKK-------------KRILSFGRKKKKP--TTDAENDASKKDVKE
          	... :    .:.  .               *: * :  ..: .   : :       . * 

MG04855.1 	DSSDEQANHGTIINLMSVDSFNVSEVTSYLHFLFASAPTQLLVSVVLLYQVLGMSAIPGF
NCU03591.1	AEADEQANLGTITTLMSVDSFKVSEVTSYLHFLIASAPTQLIVAIILLYSVMGYSAIPGF
FG09017.1 	DSNDEQANLGTIINLMSIDSFKISEVTAYLHFLCASAPTQLIVAVVLLWQVMGLSAIPGI
AN0225.1  	D-AESLANIGTIINLMAIDSFKVSEVGAYLHFLWASVPVQIVIAVTLLYRLLGFSSFAGI
          	   :. ** *** .**::***::*** :***** **.*.*::::: **: ::* *::.*:

MG04855.1 	VVMVLLLPVNIGFGRAFNSTQKKIMACSDKRIHSTNEVLQNIRIIKYFAWEHRFSESVDE
NCU03591.1	IIMALLLPVNIGFAKSFQYTQKKIMSATDKRIHTTNEVLQNIRIIKYFAWEHRFETIVDE
FG09017.1 	VVMIFLLPINYGLARGFTITSKRILAATDKRTNVTNEVLQNIRIIKYFAWEQRFGRIIDE
AN0225.1  	IIMVLMLPINLIIAKKFSKLQNQILKGTDARIHSTNEILQNIRIIKYFAWEERFQDIVNE
          	::* ::**:*  :.: *   .::*:  :* * : ***:*************.**   ::*

MG04855.1 	KRKAELKALRARYMVWACAVAVWNTVPLLITFFSFLMYTTVEKRPLHPSIAFTSISLFML
NCU03591.1	KRRAELKALRARYMVWACAVAVWNTVPILITFFTFFVYTVIEKKPLYPSIAFTAISLFML
FG09017.1 	KRQAELKALRSRFMVWALAVAIWNSVPVLITFFSFLVYTFVEHKPLKPSIAFTAISLFML
AN0225.1  	KRRAELRALRLRYILWSTAATVWYGTPILITFASFFLYTVVEKKRLTPSIAFPALSMFSL
          	**:***:*** *:::*: *.::*  .*:**** :*::** :*:: * *****.::*:* *

MG04855.1 	LRHPLDQLGDMLAHVQEAKVSIDRIEEFLSEEETDKFIQLGEDNVNEEGTRIIALKDAAF
NCU03591.1	LRYPLDQLGDMIAHVQESKVSVDRIEEFLSEEETEKYKQLGQDSVDENGNKIIGFRDASF
FG09017.1 	LRVPLDQFGDMFAHVQETKVSIDRVEEFLMEEETDKYIQLGLDNVDEDGVRRIGFKNATL
AN0225.1  	LRIPLDQLADMVAHVQESKVSLDRVDKYLNEDETEKYIQLADEDTSEP---RIALEHATL
          	** ****:.**.*****:***:**::::* *:**:*: **. :...*     *.:..*::

MG04855.1 	IWGGKDIIAA-DGSQAFRLLDIDTEFMIGKLNVIAGPTGSGKTSLLMALLGEMTLLKGRV
NCU03591.1	IWGGRNTVAK-DGSLAFRLLDLNIEFQQGKLNVITGPTGCGKSSMLMALLGEMTILKGRV
FG09017.1 	TWGSKETVSE-DSTRAFRLMDVDIDFKIGKLNIIAGPTGSGKTSMLMGLLGEMTLIEGGV
AN0225.1  	SWGTGPTNENGDKDETFRLINVDVEFLIGKLNIIAGQTGSGKTSLLMALLGEMKLLEGRV
          	 **       .*   :***:::: :*  ****:*:* **.**:*:**.*****.:::* *

MG04855.1 	YLPGGRS-REDVRPDPETGLAETCAYVAQQAWLVNANIKDNILFSARFDEKRYRDVIVAC
NCU03591.1	FLPGGRS-REDVRPDPETGLADTCAYVAQQAWLVNGSIKDNILFSAPLDEQRYKDVIVAC
FG09017.1 	YCPGGRS-REEVHPDPETGLANTIAYVAQSAWLVNANVRENILFSMPFEEQRYRDVIVAC
AN0225.1  	HLPGGTANRAELPVDTRTGLIESVAYCAQEAWLVNDTIKENIIFASPFDERRYNAVIKAC
          	. *** :.* ::  *..*** :: ** **.***** .:::**:*:  ::*:**. ** **

MG04855.1 	ALERDLEILDNGDETLVGEK0GITLSGGQKQRISLARAVYSNSKHLLLDDCLSAVDSHTA
NCU03591.1	ALERDLEILDNGDETLVGEK~GITLSGGQKQRISLARAVYSNSKHLLMDDCLSAVDSHTA
FG09017.1 	ALERDLQILDNGDETLVGEK~GITLSGGQKQRISLARALYSNSAHLLLDDCLSAVDSHTA
AN0225.1  	ALERDLEILDAGDETLVGEK~GIGLSGGQKQRISLARAIYSRARHLLLDDCLSAVDSHTA
          	******:*** ********* ** **************:**.: ***:************

MG04855.1 	QWIFNNCIRGPLMKDRTCIMVTHNIPLCVPHSDFVVVMDNGRITHQGRALEVITSGALGE
NCU03591.1	QWIFSNCIMGPLMKHRTCILVSHNIPLCVPFSDFVVQMANGRVTAQGSPQELIAAGKFGE
FG09017.1 	QWIFSNCIRGPLMKNRTCVLVTHNTTLCVPSSDYVVLLDNGRVEIQGPSAEVIASGKLGE
AN0225.1  	KHIFREALSGPLMLNRTCILVTHNIALTAPYADFIVALDNGKVSFQGRPDEAGASKALGE
          	: ** :.: **** .***::*:** .* .* :*::* : **::  ** . *  ::  :**

MG04855.1 	EIAQKAKSETPNISRIPSRVPS-SVGEGSGNTLL-DTDGDDHLSK-------PKNAKKAK
NCU03591.1	DLAQKSYPGSAHVSRVPSRVPS-SVGEEGDHTMINEADGSESQTK-------AQTIKKEH
FG09017.1 	EI-QKTQPGSANPSRIPSRVPS-SVGDD-ETTVNGQGDALDGVDS-------SKDTKSKP
AN0225.1  	ELAKSRPGSRSSSSPPRSRRPSNSANDDNESHANGIPNGTANGTANGAVNGTPGKTLQKT
          	::::.     .  *   ** **.*..: .     .  :.      ..: ..:.    .  

MG04855.1 	K-AEAMEETKATGAVKWPVMKLYLASMGSWWFWVVAGCIFISQQASDVVSNLWIKQWASQ
NCU03591.1	KKQDAMEESKATGAVKWPVMKLYLTAMGKWWFWVLSAVVFGAQQFSGVATSLWIKEWANQ
FG09017.1 	K-KDAMEETKATGSVKWPVLKLYLQAMGPWWFWVVALFVFVAQQVSGLATNLWVREWANQ
AN0225.1  	ATSKKHAETKAVGSIKWPIIKMYLFSMGPWYYWIVAVLVFTLQQLGSVSTNIWIRQWANS
          	 . .   *:**.*::***::*:** :** *::*:::  :*  ** ..: :.:*:::**..

MG04855.1 	YTT-EVSSVPISTSSHMYGTQSFAPTYMVPVQTYVKDQLA-RNGNATALDIVVNSEVNAQ
NCU03591.1	YAMDEVVAPTFNMNSQSYSSQTVSPTYFASIATYIK------GNDSNTFSTTSNSDVNVG
FG09017.1 	YVE-QGGPIGMEVKSNAYSTSQFSKVSFANAVRFGSADDTPEQLNGAIFMQAGQENVNAM
AN0225.1  	YSA-DNVDINSEGTYAAMAHFKFPSFNVLSVPRTSG-----VAPQIGTQTVDSDNNVNVT
          	*   :      . .    .   ..   .                :        :.:**. 

MG04855.1 	YYLVVLAIIGLAGSLTAFLRDLWIFFGSLTASAKIHTRLLNTVTRAKFRFFDVT0PLGQM
NCU03591.1	YYLTMLAIIGIGGAVAAFARDMWIFFGSLTASWKLHQRLMRAVAGARFKFFDVT~PLGQI
FG09017.1 	YYLTVLAVIGIAGALAALFRDLWVFLGSLTASEHLHNRLIASVTRAKFKFFDVT~PLGQL
AN0225.1  	YYLGIYALLGVLYIAISATREGVLFWGSLHASNSIHKRLLKTVMHAKFKFFDST~PLGQL
          	*** : *::*:     :  *:  :* *** **  :* **: :*  *:*:*** * ****:

MG04855.1 	MNRFSKDMEAVDQEVAPIAIGILSCALGITVTVVLIASITPGFLIAAVFITIAYVLLAKF
NCU03591.1	MNRFSKDLEAIDQEVAPIAIGVMSCVLGIGVTVTLIAWITPGFLIAGFFITILYVFLGAF
FG09017.1 	MNRFSKDLEAVDQEIAPVAIGVISCGISLGMTVGLITYITPKFLYAAAGISVLFYLIAAF
AN0225.1  	MNRFSKDVEAIDQEVAPVAIGMLHSLASVVMIVILISIITPGFLIAAVFITMVYFALGAI
          	*******:**:***:**:***:: .  .: : * **: *** ** *.  *:: :  :. :

MG04855.1 	YLASSRDLKRLESVQRSPLFQQFGETLS2GVTTIRAYGDERRFVRDNLTRINGQLRPMIY
NCU03591.1	YLHASRDLKRLESVQRSPLFQQFGETLS~GVVTIRAYGAERRFIRENFAKINAQIRPFIY
FG09017.1 	YLRASRDLKRLEAVQRSPLFQQFGETLS~GMTTIRAYGDERRFIRDNLEKVNVQSRPFIY
AN0225.1  	YLNASRDMKRLESVQRSPLYQQFGETLN~GIVTIRAYGDGPRFLVDNHRRINNYNRPHIY
          	** :***:****:******:*******. *:.******   **: :*  ::*   ** **

MG04855.1 	LWATNRWLAFRTDLLGDFVSFFAGVFVILSIGVIDAGWAGISLSYAIGFAENILWLVRLY
NCU03591.1	LWAANRWLAFRTDVLGDLVAFSAGVFVILSLGKIDPGSAGISLSYAIGFADNILWLVRLY
FG09017.1 	LWACNRWLAFRADLVGDLVAFFAGMFLILNLGKTDAGAAGISLSYALSFTESVLWLVRLY
AN0225.1  	LWAANRWLAFRVDITGALVSFLTATFILSNIGRIDAGAAGLSLTYAVTFTENVLWLVRLY
          	*** *******.*: * :*:* :. *:: .:*  *.* **:**:**: *::.:*******

MG04855.1 	SINEQNMNAVERIKEYLEVEQEAAPICEKNRPPQNWPAQGSVEFINYTTSYRKELDPVLR
NCU03591.1	AMNEQNMNSVERIKEYLDVEQEAVAVVEDNRPPANWPAHGSVEFINYTTRYRAELEPVLR
FG09017.1 	AINEQNMNSMERIKEYLDVEQEADAIMEDSRPPVDWPQKGSVEFVDYTTSYRKELSPVLQ
AN0225.1  	SEVQQSMNSVERVKEYLEVEQEAPPVIADSRPPPNWPSKGSVQFSNYSTRYRLDLDPVLR
          	:  :*.**::**:****:***** .:  ..*** :** :***:* :*:* ** :*.***:

MG04855.1 	NVTFKISPQEKVGIVGRTGAGKSSLALAIFRALEADGGKILIDGIDIGLIGLRDLREAIT
NCU03591.1	NLTFKIEAHEKIGIVGRTGAGKSSLTLAIFRALEADEGKILIDDIDIGLIGLRDLREALT
FG09017.1 	NITLKIEPQEKVGIVGRTGAGKSSLALAIFRALEADEGKILIDGLDIGSIGLQDLRENIT
AN0225.1  	DVSFTVQSGEKVGIVGRTGAGKSSLALALFRGLEAETGRITIDGVDIGTIGLKDLREAIT
          	::::.:.. **:*************:**:**.***: *:* **.:*** ***:**** :*

MG04855.1 	IVPQEPTLFTGTIRSNLDPFHLFTDEQIYKSLQRVQLIG---PDETIPTADASPVLPASP
NCU03591.1	IVPQEPTLFMGTIRTNLDPFNMYTDEEIFAALRRVHLIK---ADEVIPGAVSTALLPQTP
FG09017.1 	IVPQDPTLFHGSIRSNLDPFDQYTNDQIFAALRRVQLIG---PDE----PATSPPTPTVP
AN0225.1  	IVPQDPTLFTGTIRSNLDPFGHFTDEQIFTAIRRVHLIGSGTSGTATPVTASTLTVTEQG
          	****:**** *:**:*****  :*:::*: :::**:** :.:..   . . ::   .   

MG04855.1 	TTPGG---------ASNKNIFLNLSSKVSESGSNLSQGQRQLLCLARALLKEPRVLVMDE
NCU03591.1	VAPESESEPSRPATATNKNIFLDLSSPVTESGNNLSQGQRQLLCLARAMLKKPTVLVMDE
FG09017.1 	DSPTA---------KTNQNIFLNLESPVAASGTNLSQGQRQLLCLARAMLKSPTVLVMDE
AN0225.1  	SASPSEGNAISSTVLDNKNIFHNLESPVSESGSNLSQGQRQLLCLARALLKNPKVLMMDE
          	 :. .....  .:   *:*** :*.* *: **.***************:**.* **:***

MG04855.1 	ATASIDYATDSKIQDTIREMKDTTIITIAHRLQTIADYDKVLVLDKGEVVEYAHPWELMR
NCU03591.1	ATASIDYATDAAIQETIRELTS-TIITIAHRLQTIADYDKVLVLDKGKIVEYAHPWELMQ
FG09017.1 	ATASIDYATDSKIQETIRELTG-TVITIAHRLATIVDYDKVLVLDKGQVVEYAHPWELIN
AN0225.1  	ATASIDYTTDAKIQETLRELRDNTIITIAHRLQTIIDYDKVLVLDHGRVVEFDHPWTLVN
          	*******:**: **:*:**: ..*:******* ** *********:*.:**: *** *:.

MG04855.1 	KGEGGSFKSMCDMSGDTELLAKAAKKAFDAKKLIDVDDEAADAAP---------------
NCU03591.1	KTDG-SFRSMCEMSGDYDTLVKIAKKAYKTKQLVDIDEEDEQPSTATTAEGSGTGDEGKK
FG09017.1 	KKEG-TFRSMCDMSGELDVLLKAAKKKWDSGRLVDDS-----------------------
AN0225.1  	KEGG-IFRSMCENSGNLETLLELAKKAWEQKRLVDDS-----------------------
          	*  *  *:***: **: : * : *** :.  :*:* .                       

MG04855.1 	----
NCU03591.1	DGEN
FG09017.1 	----
AN0225.1  	----
          	
```
